# Advances in wildlife abundance estimation using pedigree reconstruction

**DOI:** 10.1002/ece3.10650

**Published:** 2023-10-18

**Authors:** Elias Rosenblatt, Scott Creel, Katherina Gieder, James Murdoch, Therese Donovan

**Affiliations:** ^1^ Vermont Cooperative Fish and Wildlife Research Unit, Rubenstein School of Environment and Natural Resources University of Vermont Burlington Vermont USA; ^2^ Department of Ecology Montana State University Bozeman Montana USA; ^3^ Vermont Fish and Wildlife Department Rutland Vermont USA; ^4^ Wildlife and Fisheries Biology Program, Rubenstein School of Environment and Natural Resources University of Vermont Burlington Vermont USA; ^5^ U.S. Geological Survey, Vermont Cooperative Fish and Wildlife Research Unit, Rubenstein School of Environment and Natural Resources University of Vermont Burlington Vermont USA

**Keywords:** abundance, density, pedigree reconstruction, population monitoring, simulation

## Abstract

The conservation and management of wildlife populations, particularly for threatened and endangered species are greatly aided with abundance, growth rate, and density measures. Traditional methods of estimating abundance and related metrics represent trade‐offs in effort and precision of estimates. Pedigree reconstruction is an emerging, attractive alternate approach because its use of one‐time, noninvasive sampling of individuals to infer the existence of unsampled individuals. However, advances in pedigree reconstruction could improve its utility, including forming a measure of precision for the method, establishing required spatial sampling effort for accurate estimates, ascertaining the spatial extent of abundance estimates derived from pedigree reconstruction, and assessing how population density affects the estimator's performance. Using established relationships for a stochastic, spatially explicit simulated moose (*Alces americanus*) population, pedigree reconstruction provided accurate estimates of the adult moose population size and trend. Novel bootstrapped confidence intervals performed as expected with intensive sampling but underperformed with moderate sampling efforts that could produce abundance estimates with low bias. Adult population estimates more closely reflected the total number of adults in the extant population, rather than number of adults inhabiting the area where sampling occurred. Increasing sampling effort, measured as the proportion of individuals sampled and as the proportion of a hypothetical study area, yielded similar asymptotic patterns over time. Simulations indicated a positive relationship between animal density and sampling effort required for unbiased estimates. These results indicate that pedigree reconstruction can produce accurate abundance estimates and may be particularly valuable for surveying smaller areas and low‐density populations.

## Graphical abstract

Pedigree reconstruction is an emerging, attractive alternate approach to estimating abundance because its use of one‐time, noninvasive sampling of individuals to infer the existence of unsampled individuals. We use a stochastic, spatially explicit simulated moose population to estimate the precision, bias, and accuracy of pedigree reconstruction as an abundance measure using two sampling approaches across variable population densities. These results indicate that pedigree reconstruction can produce accurate abundance estimates and may be particularly valuable for surveying smaller areas and low‐density populations.

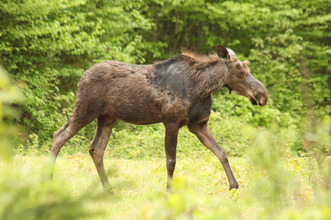

## 1 INTRODUCTION

Precise and unbiased estimates of abundance, growth rate, and density are often critical for developing effective wildlife management strategies, especially for threatened and endangered species (Williams et al., 2002). However, such estimates can be difficult to obtain (Ripple et al., 2015). Many species inhabit remote areas where terrain and vegetation conditions make surveying difficult. Some species also avoid humans or are secretive, limiting direct observation (Morellet et al., 2007; Ripple et al., 2015). Abundance estimates generally must account the inability of observer(s) to detect all individuals, as complete, unbiased counts are rarely possible. However, methods to estimate population size are often costly and, in many cases, impractical due to constraints on time, funding, and logistics (Morellet et al., 2007; Rabe et al., 2002). As many wildlife populations face threats from a changing climate and other anthropogenic pressures (Ripple et al., 2015), improved abundance estimators would be beneficial, particularly for species and populations that are difficult to survey.

There are several methods of estimating population size, trend, and density, and their applications represent trade‐offs in the spatial scale surveyed, effort (and cost) required, and precision of abundance estimates. For species that are not easily individually identifiable, raw counts of individuals made from the air‐ or ground‐based transects are adjusted to account for detection probability with sightability (Bontaites et al., 2000; Samuel et al., 1987) or distance sampling models (Buckland et al., 1993). These survey methods can be used to monitor populations across large areas, but often are prohibitively expensive to conduct regularly (Cook & Jacobson, 1979; Rabe et al., 2002) and may not provide estimates precise enough of abundance for low‐density or cryptic species that are useful for their conservation (Olson et al., 2005). For species in which individuals can be uniquely distinguished (i.e., through distinctive physical characteristics, researcher marks like tags or collars, or genetic markers), capture–mark–recapture (CMR; Otis et al., 1978; Pollock & Otto, 1983) and, more recently, spatial capture recapture (SCR; Efford, 2004; Borchers & Efford, 2008) models are common methods for estimating abundance. These methods use sighting data of individuals over repeated surveys to estimate abundance while accounting for imperfect detection (Pollock & Otto, 1983). These methods require repeated surveys to collect observation data, which can increase costs and still may not yield enough information to identify population trends, particularly for wide‐ranging, low‐density species (Lukacs & Burnham, 2005; Royle et al., 2018). Most recently, close‐kin capture–mark–recapture methods relax the assumption of “recapture” to “recapture of close kin”, opening new avenues of population estimation using samples from both living and dead individuals (Bravington et al., 2016; Marcy‐Quay et al., 2020). Finally, abundance methods using animal sign or public sightings reduce costs and effort for data collection, but generally yield lower precision compared to measures of abundance that account for detection (Härkönen & Heikkilä, 1999; Rönnegård et al., 2008).

**FIGURE 1:**
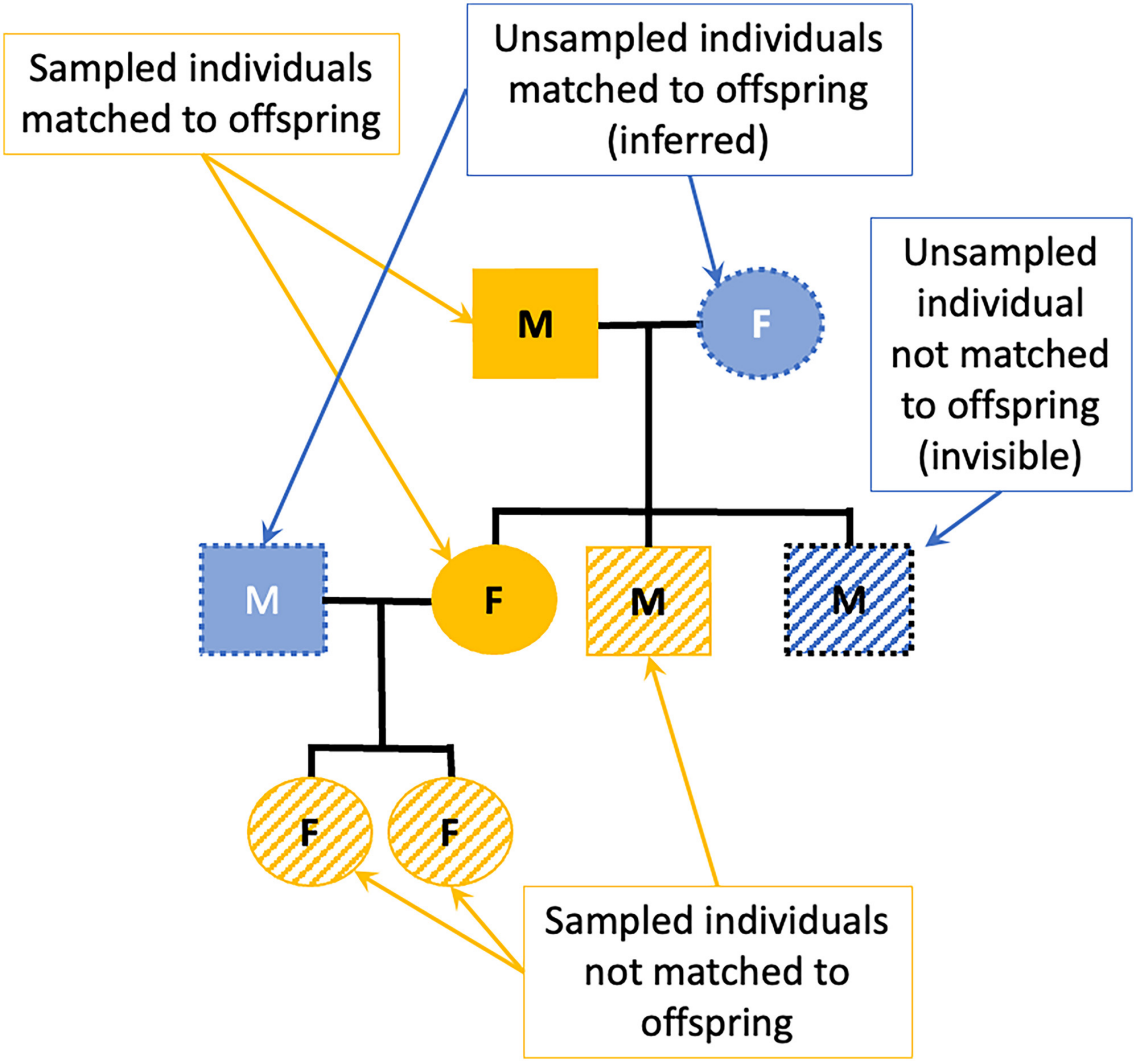
An example pedigree reconstructed from genetic data that identified females (circles), males (squares), and their familial relationships (black lines). Individuals may be genetically sampled (gold) or unsampled (blue), and genetically matched (solid) or unmatched (patterned) to sampled offspring. Pedigree reconstruction allows the inference of unsampled individuals by genotypic data from offspring and mate (inferred individuals; blue, solid), and of unsampled individuals that have not been matched to offspring (invisible individuals; blue, patterned).

Pedigree reconstruction is an emerging contribution to the growing toolkit of abundance estimation methods (Arandjelovic & Vigilant, 2018) and a useful approach for estimating abundance of low‐density, cryptic species that are otherwise difficult to study. Pedigree reconstruction uses genetic data collected from many individuals to create a pedigree for the population (Figure 1). Pedigrees are used for a variety of conservation applications (Blouin, 2003) as they provide valuable population genetics measures related to inbreeding (Jones et al., 2002; Liberg et al., 2005) and effective population size (Luikart et al., 2010). Pedigree reconstruction also infers the existence and genotypes of individuals that are not directly sampled, which allows for estimates of population size. This is possible when an offspring and only one of its parents are sampled—in this case, another (unsampled) parent must exist as a portion of its genetic information is contained in the offspring's genotype. By combining data on relationships between sampled individuals and inferred unsampled individuals, pedigree reconstruction can provide an abundance estimator by correcting the count of sampled and inferred individuals with these “invisible” individuals (Creel & Rosenblatt, 2013; Figure 1).

The prospect of estimating abundance using pedigree reconstruction is an active area of inquiry (Creel & Rosenblatt, 2013; Ekblom et al., 2021; Hettiarachchige & Huggins, 2018; Larroque & Balkenhol, 2023; Nielson et al., 2001; Skaug, 2001, Spitzer et al., 2016). The approach is attractive because it can use noninvasive genetic samples and only requires individuals to be sampled once (in contrast with genetic CMR studies). Creel and Rosenblatt (2013) successfully validated this approach with a simulated African lion (*Panthera leo*) population. Spitzer et al. (2016) then applied this pedigree reconstruction approach to two brown bear (*Ursus arctos*) populations and found that pedigree reconstruction estimates were comparable to existing CMR estimates. Larroque and Balkenhol (2023) used simulated wild boar (*Sus scrofa*) and red deer (*Cervus elaphus*) populations to compare pedigree reconstruction estimation with CMR and alternative abundance estimators reliant on identifying kinship relationships. Larroque and Balkenhol (2023) demonstrated the pedigree reconstruction was precise and insensitive to population fecundity, but cautioned its accuracy is highly dependent on knowing roughly how many individuals should be sampled. With further development, the pedigree reconstruction method could provide affordable and efficient abundance estimates for wildlife studies.

While pedigree reconstruction is an attractive alternative to other commonly used methods, its application can be broadened by addressing known limitations. First, pedigree estimation has been developed to produce point estimates of abundance, but no measure of precision has been established for this method, nor has its ability to estimate population growth rates been evaluated (Spitzer et al., 2016). Second, the sampling effort required for pedigree estimation has been evaluated only in terms of sampling a proportion of unsampled individuals in a target population (Creel & Rosenblatt, 2013). However, individuals are distributed nonrandomly in space, and at the time of sample collection the identity of individuals is unknown. Practically speaking, the study design must consider the spatial dynamics of a population such as home range size and habitat use to maximize the number of uniquely sampled individuals under consideration. Finally, the relationship between the accuracy of estimates from pedigree reconstruction and population density is unknown yet crucial. Pedigree reconstruction may meet a need for a noninvasive, efficient, flexible, and affordable approach to monitoring populations for a variety of species. However, these critical questions must be tested with spatially explicit, simulated populations along with comparisons with established abundance measures in well‐studied populations to better inform applications of this method with wild populations.

**FIGURE 2:**
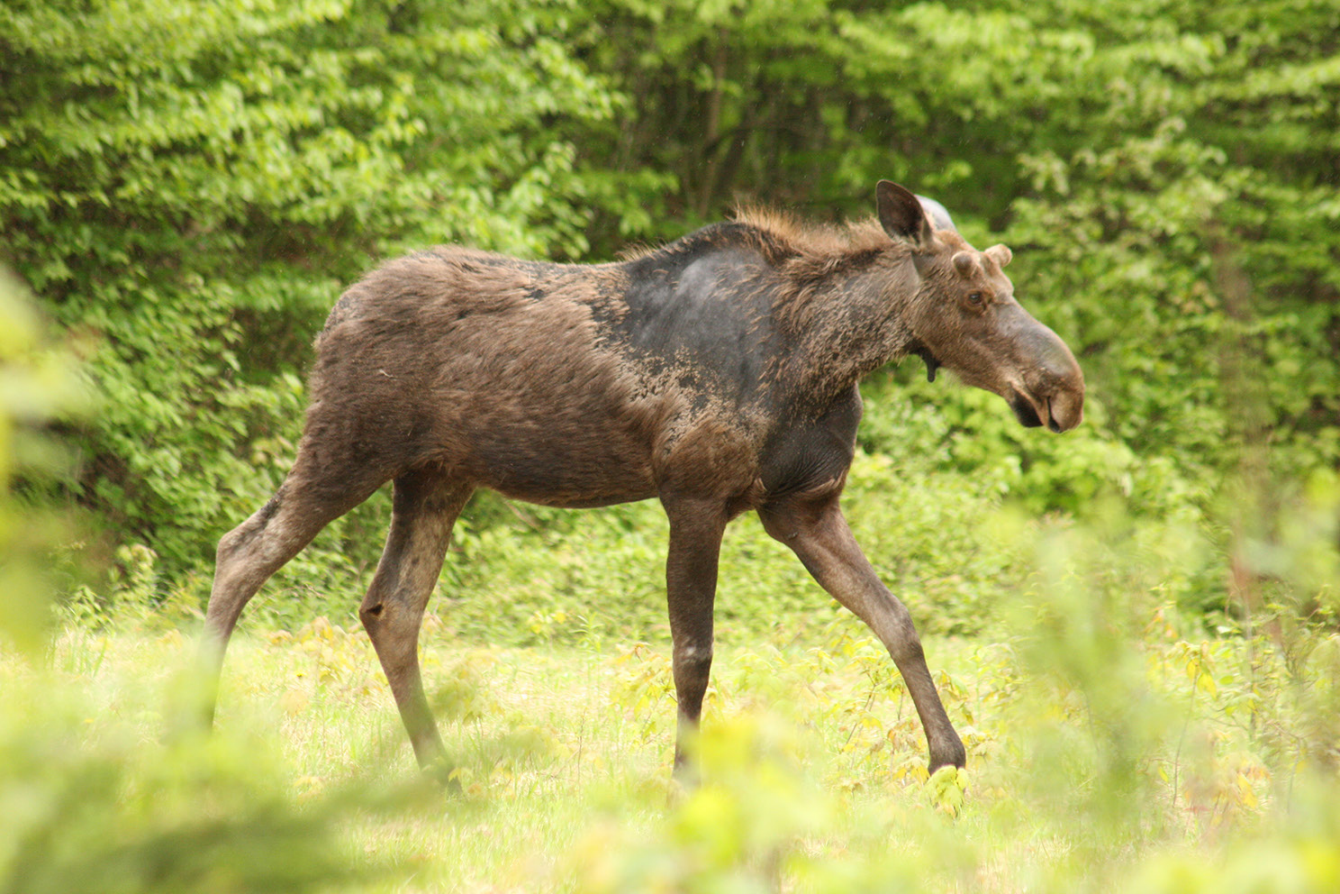
An adult male moose (*Alces americanus)* shedding its winter coat in Vermont, USA. Photograph by E. Rosenblatt.

In this study, we enhanced the Creel and Rosenblatt (2013) pedigree reconstruction population estimator by integrating a Bayesian, probabilistic approach to estimate population size along with a measure of uncertainty. We used a stochastic, spatially explicit simulated moose (*Alces americanus*; Figure 2) population to (1) evaluate the accuracy of an updated pedigree reconstruction approach to estimate abundance and growth rates with varying sampling efforts and (2) develop a location‐based, noninvasive sampling design that mimics a realistic field study and identify the effort level required for accurate abundance and growth rate estimates. We then (3) investigate the relationship between the bias and precision of pedigree reconstruction abundance estimates and population density. This study advances a promising method for estimating abundance and strengthens its capacity for application to wildlife populations.

## 2 METHODS

### 2.1 Pedigree reconstruction population estimator

A population of adult individuals can be partitioned into four segments, as defined by pedigree reconstruction (Appendix 1: A1 and A2). The first two segments include individuals that are genetically sampled and are either related to other sampled individuals (parent–offspring relationship derived from a reconstructed pedigree; *N*_linked_), or unrelated to any other sampled individual (*N*_unlinked_). The third segment includes individuals that are missed by genetic sampling efforts but have left evidence of their existence in the population because they have successfully reproduced (*N*_inferred_). These unsampled individuals are inferred if their mate and at least one of their offspring have been sampled. The fourth segment includes individuals that are both missed by genetic sampling and have no evidence of successful reproduction (and thus cannot be inferred) and are invisible to pedigree reconstruction (*N*_invisible_). An estimate of the adult population size is the sum of these four components (Equation 1), which can be estimated by computing the conditional probabilities based on the three known terms and is an improvement of the original Creel and Rosenblatt (2013) estimator (Appendix 1: A1). This modification of pedigree reconstruction as a means of population estimation follows the logic of basic CMR approaches in which a population estimate is obtained by dividing the known sample by an estimate of capture probability (Williams et al., 2002). Importantly, only adult individuals capable of breeding are considered in these four conditions, whereas immature individuals help identify adult individuals as linked or inferred individuals. Population estimates from pedigree reconstruction should therefore be interpreted as the size of the adult segment of a population.

Although the approach provides a method for estimating total population size, it lacks integration of uncertainty around parameter estimates, which could be sizeable in cases where too few individuals are sampled (Spitzer et al., 2016). Assuming high confidence in the ability of genetic information to determine relationships and infer the existence of unsampled individuals, uncertainty in the estimates from pedigree reconstruction lies in the estimation of the marginal probabilities (*p*_sampled_ and *p*_matched_). Observed numbers of individuals used to calculate these marginal probabilities are subject to sampling effort and study design, and therefore represent a random process that introduces uncertainty. We propose that a beta distribution can be used to set prior probabilities on the *p*_sampled_ and *p*_matched_. The observed data, derived from pedigree reconstruction, can then be used to update vague prior distributions to posterior distributions, which in turn can be used in bootstrapping to generate the joint probabilities and population estimates across multiple trials (Appendix 1: A2). In the bootstrap, random values of both marginal probabilities can be drawn and multiplied together to calculate a random sample of joint probabilities. These joint probabilities can be used to produce a random sample of likely population estimates, from which confidence intervals can be obtained that provide measures of uncertainty (Appendix 1: A2).

As with any method of estimating population size, there are several assumptions that must be met when using pedigree reconstruction. First, the probability of being sampled (*p*_sampled_) should not differ between matched and unmatched individuals. *p*_sampled_ is calculated from the proportion of linked individuals that were sampled (Appendix 1: A1); there is no means of estimating *p*_sampled_ for unmatched individuals, as the number of “invisible” individuals is unknown. For the same reason, the probability of being matched to another individual (*p*_matched_) should not differ between sampled and unsampled individuals. Second, this approach considers only sexually mature adults in calculating conditional and joint probabilities but requires genetic samples from all age classes to identify adults as linked, unlinked, or inferred. Similarly, population size estimates correspond to the total adult population size, which can easily be corrected to include juveniles if age distributions are known for the population. Third, mortality must be accounted for when reconstructing the pedigree, and should not include samples from deceased individuals other than to infer individuals. If not accounted for, pedigree reconstruction will accumulate individuals no longer present in the population in its calculation of total population size, resulting in an overestimation of abundance (Creel & Rosenblatt, 2013). Creel and Rosenblatt (2013) presented approaches to account for mortality in pedigree reconstruction, primarily by applying available mortality rates to sampled individuals over multiple years of sampling. Fourth, sample collection must allow the designation of individuals as a juvenile or an adult. Finally, all adults must exist in one of the four states described above; additional states can lead to biased estimation of both marginal probabilities.

### 2.2 Applying updated pedigree reconstruction to a simulated moose population

**FIGURE 3:**
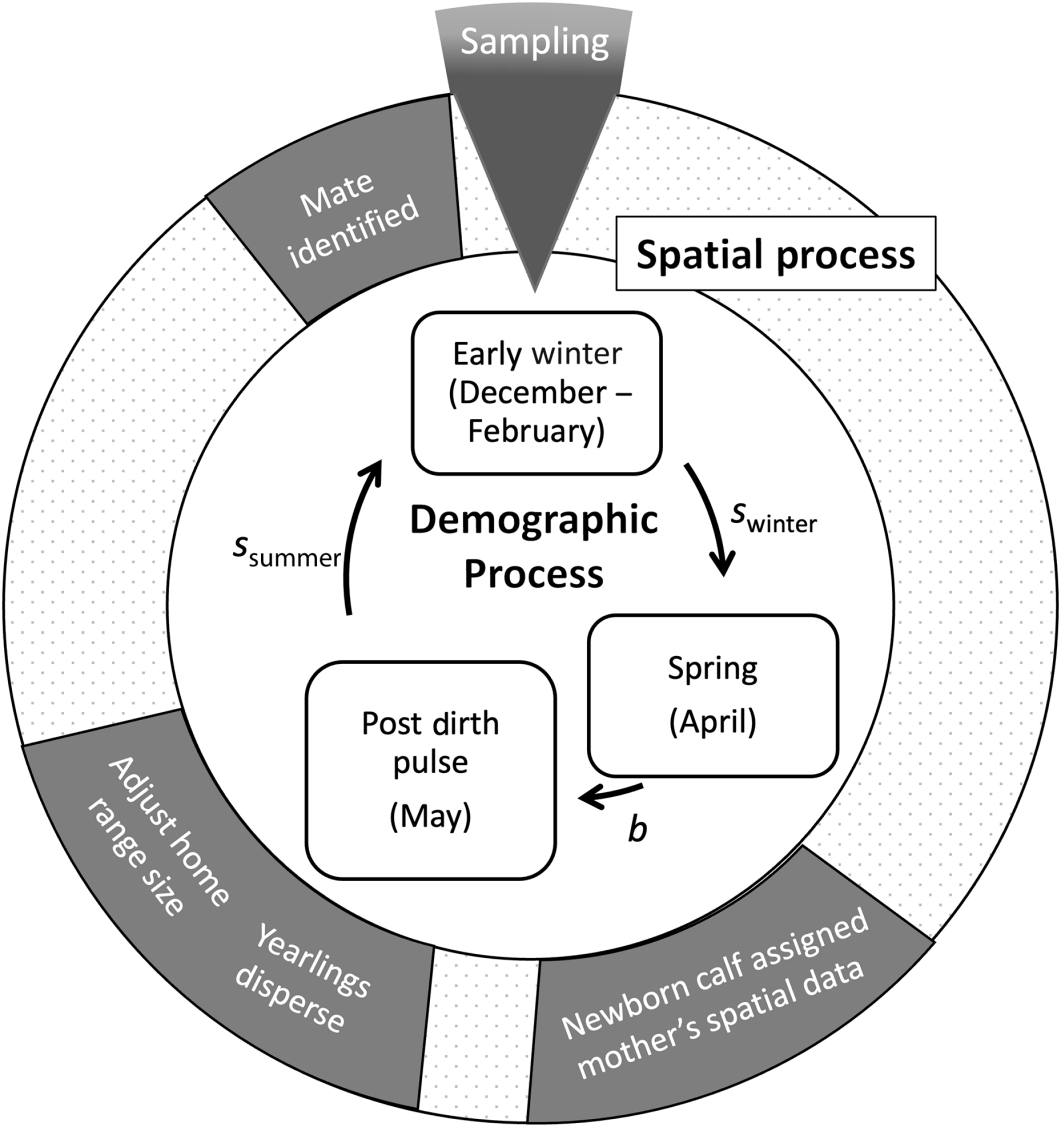
A summary of the processes involved with each time step (year) of the simulated moose (*Alces americanus*) population. Demographic processes shown (inner cycle) include seasonal population sizes (boxes) influenced by age‐specific survival rates (s), age‐specific birth rates (b), and timing of the population's birth pulse. Spatial dynamics shown (middle cycle) include the timing of mate selection, yearling dispersal, and home range adjustment with aging. Sampling focused on the population in early winter, prior to winter mortality.

**FIGURE A1:**
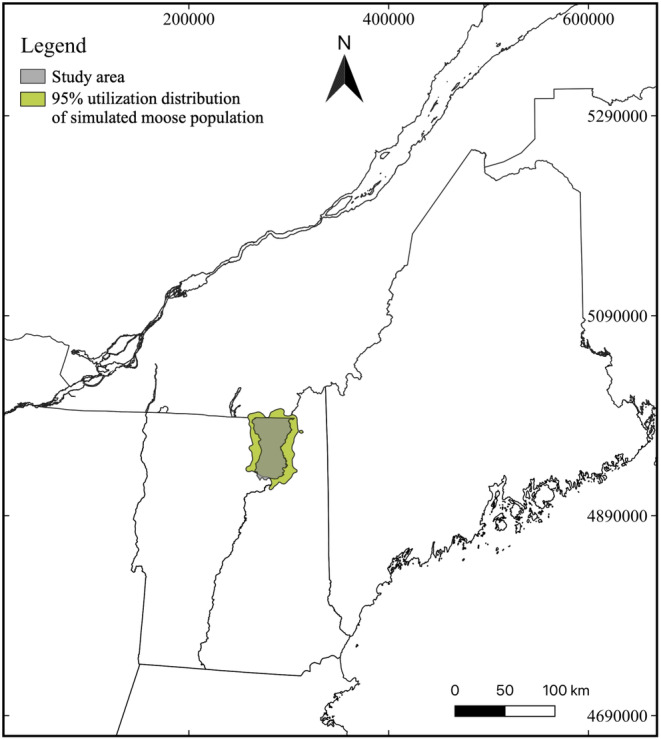
Study area in Vermont, USA (1650 km^2^; gray polygon) where we simulated a spatially explicit moose *(Alces americanus)* population and various genetic sampling schemes. Simulated moose ranged primarily around the study area (green polygon), though dispersals did occur beyond this area.

To evaluate the performance of pedigree reconstruction, we used an individual‐based model (Grimm, 2019) to simulate a moose population. Moose are a candidate species for the use of pedigree reconstruction as they often inhabit remote, rugged areas with dense vegetation and spend much of the year in solitude or small groups (Ballard et al., 1991; Harris et al., 2015). We simulated the population dynamics of a stable moose population over 25 years in a 1650‐km^2^ management unit in northeastern Vermont (Figure A1). Each year of our simulation began at the beginning of winter (December) and included the annual processes that a moose experiences in the wild, including a winter survival (February–April), synchronous birth pulse and dispersal (May), and summer survival (May–December; Figure 3). Samples (e.g., scat) for pedigree reconstruction were collected during winter. We emphasize that our goal was not to study this specific population in Vermont. Rather, our aim was to apply the dispersal, birth, and death of individuals using realistic, empirical rates from multiyear studies of moose in the region to develop a known pedigree and derive population estimates for the population.

### 2.3 Individual‐based model simulation

We used demographic data available for the moose populations, available from radio‐collar studies across the region, adjusted to ensure stable population growth (Table 1; Ellingwood et al., 2020). Our individual‐based model simulation was conducted in R, and source code is available in the Data Availability statement (R Core Team, 2021). We incorporated senescence (Gasaway et al., 1983) in our simulated population by increasing the odds of mortality by 1.6‐fold every year after 9 years of age (Ericsson & Wallin, 2001) and ceasing reproduction in adult females after 14 years of age (Ericsson et al., 2001). We used age‐specific vital rates until 15 years for both sexes, after which individuals survived and reproduced at a constant rate (Table 1). Birth rates, expressed as newborn calves per cow per year, rapidly increased with age, reaching a maximum rate for adults aging from 3 to 14 (Table 1). Twinning is rarely observed in Vermont and other New England states (DeBow, 2020), so we limited birth rates to a single newborn calf per adult female, per year. These vital rates corresponded to a stable, slightly positive population trend (λ_asymptotic_ = 1.003), estimated using the popbio package in R (R Core Team, 2021; Stubben & Milligan, 2007). Our simulated population began with 659 individuals with a stable age distribution (Table 1; R Core Team, 2021; Stubben & Milligan, 2007).

**TABLE 1:** Vital rates by sex, age, and season, and yearling dispersal distances used in the spatially explicit simulated moose (*Alces americanus*) population. Vital rates and dispersal distances were based on moose multiyear studies in Vermont and New Hampshire, USA (Ball, 2017; Ellingwood et al., 2020). Reduction in survival rates for older age classes were included to account for adult senescence (Gasaway et al., 1983).

Sex	Age	Winter survival	Birth rate	Summer survival	Yearling dispersal distance (SE)	Segment of starting population (*n*; *N* = 659)
Female	0 (Calf)	0.70	0	0.70	–	72
	1	0.93	0.07	0.95	2.3 km (0.4)	47
	2	0.93	0.77	0.95	–	42
	3–9	0.93	0.90	0.95	–	180
	10	0.89	0.90	0.92	–	15
	11	0.84	0.90	0.88	–	12
	12	0.76	0.90	0.82	–	9
	≥13	0.67	0.90	0.74	–	11
Male	0 (Calf)	0.70	–	0.70	–	72
	1	0.88	–	0.90	9.3 km (3.1)	45
	2–9	0.88	–	0.90	–	142
	10	0.82	–	0.85	–	5
	11	0.74	–	0.78	–	4
	12	0.64	–	0.69	–	2
	≥13	0.53	–	0.58	–	1

We incorporated spatial movement and home range establishment based on data available from studies in Vermont and neighboring New Hampshire (Ball, 2017; Blouin et al., 2021a). Individuals in our simulated population were assigned random home range centroids within the study area using the spsample() function in the sp package (Bivand et al., 2013; Pebesma & Bivand, 2005). We assigned locations based on global positioning system (GPS) collar data collected on moose of the same sex and age in the study area, adjusted to be relative to each simulated animal's home range centroid (Blouin et al., 2021a, 2021b). Animal centroids would change as individuals dispersed, and location data would be updated as individuals aged into older age classes. We integrated available GPS collar data to allow for realistic variation in home range size and shape, and to establish both winter and annual home ranges. We used annual home ranges to determine mate selection and the use of the study area, while we used winter home ranges to develop spatially explicit sampling for pedigree reconstruction.

In each year of the simulation, individuals began with established centroids and home ranges, and survived the winter at age‐specific rates (Table 1) resulting from a Bernoulli process (the rbinom() function in R). Surviving females (>1 year old) then reproduced in the spring (May) with a random draw from a Bernoulli distribution based on their age‐specific reproductive rate (Table 1). Reproducing mothers were randomly assigned a mate from a list of candidate fathers (>3 years old) that were alive the previous breeding season (fall) and overlapped spatially. We determined overlap using 100% minimum convex polygons calculated using the adehabitatHR package in R (Calenge, 2006). Offspring were added to the population, with established relationships to their parents. Newborn calves had home ranges that matched their mothers until they dispersed during the following year (Ballard et al., 1991). At this time, all individuals in the population aged 1 year. New yearlings would then disperse at a random bearing (0–359) and distance drawn from a normal distribution derived from available estimates of dispersal distances (Table 1). Individuals then survived the summer to the end of the annual time step at age‐specific rates (Table 1) using a Bernoulli process.

### 2.4 Genetic sampling of simulated individuals

We simulated noninvasive genetic sampling from fecal collection of the population in early and mid‐winter months (December–February) to determine individual identity, sex, and parent–offspring relationships. We assumed demographic closure during this period, which is appropriate as the early winter months precede the period of greatest winter mortality (March–April; DeBow et al., 2021). Importantly, we assumed that a study of this nature could determine the basic age class (calf or adult) of an individual at collection from track and fecal pellet size (Franzmann & Schwartz, 2007; Koitzsch et al., 2022). Again, calves allow the identification of matched, sampled adults and matched, unsampled adults, but are not included in the adult population estimate (Appendix 1: A1). We determined that if a cow with a dependent calf (<1 year old) was sampled, the calf would be sampled as well. We note that this determination did not violate the assumption that the probability of being sampled does not differ between matched and unmatched individuals. The presence (or absence) of calf sign should not influence the probability of its mother being sampled, as cow–calf pairs travel often in each other's tracks or in close proximity during the snowy, winter months considered in this study.

After a 20‐year simulation period that established parent–offspring relationships, we simulated genetic sampling for moose over 5 years to test the accuracy of the updated pedigree reconstruction estimator. In each year, previously unsampled individuals were added to the pedigree, broadening and lengthening the pedigree with each passing generation. We did not incorporate duplicated samples from the same individual. We did not incorporate uncertainties in pedigree reconstruction, and assumed that parent–offspring assignments were accurate.

### 2.5 Objective 1: Accuracy of updated pedigree reconstruction

In every year of sampling, we randomly sampled animals that existed in the study area at a range of sampling intensities over 100 iterations: 10%–90% of unsampled individuals, in 10% increments, resulting in 900 sampling iterations each year; referred hereafter as population‐based sampling (Table 2). We calculated the number of live adults that were linked (sampled and matched to an offspring), unlinked (sampled and unmatched to an offspring), and inferred (unsampled, but matched to an offspring) for each iteration (Appendix 1: A1). We then estimated *p*_sampled_ and *p*_matched_, and estimated adult population size based on Equation 4 in Appendix 1: A1. We evaluated the precision, bias, and accuracy of these estimates using coefficient of variation (CV), scaled mean error (SME), and scaled root mean squared error (SRMSE), respectively, relative to the known adult population size inside of the 1650 km^2^ study area. We also used these population estimates to estimate annual population growth rate and compared these growth rate estimates to the true annual population growth rate. We also tested the ability of bootstrapping to estimate uncertainty around each population estimate. For each sampling iteration, we calculated the 95% confidence intervals using the approach described in Appendix 1: A2 and reported the proportion of these iterations that contained the known population size.

**TABLE 2:** Average number moose sampled in each year of sampling a simulated moose (*Alces americanus*) population. Moose of all ages were available for sampling, but pedigree reconstruction estimates the number of adults in this simulated population. Sampling efforts for spatially based sampling are thinned for brevity.

Year		1	2	3	4	5
Population size		767	771	773	796	828
Adults		597	605	588	604	638
Sampling effort		Mean number of new individuals sampled (range)				
Population‐based sampling	10%	105 (76–131)	92 (69–122)	86 (55–117)	83 (58–109)	79 (59–102)
	20%	201 (162–236)	159 (116–202)	133 (106–169)	125 (103–155)	111 (84–139)
	30%	293 (259–335)	202 (152–246)	161 (130–194)	137 (105–169)	131 (98–158)
	40%	380 (346–411)	225 (190–259)	167 (134–194)	147 (116–169)	142 (115–169)
	50%	459 (432–495)	237 (206–271)	169 (141–192)	155 (131–176)	151 (126–173)
	60%	536 (501–571)	233 (195–261)	171 (150–192)	162 (135–194)	160 (136–182)
	70%	604 (583–633)	221 (189–246)	170 (146–188)	171 (148–195)	169 (147–187)
	80%	664 (635–688)	208 (185–242)	173 (154–195)	179 (158–194)	179 (161–200)
	90%	717 (700–735)	188 (170–207)	179 (169–194)	186 (174–201)	186 (175–199)
Spatially based sampling	10 km^2^	66 (46–87)	59 (39–85)	53 (30–78)	51 (31–73)	54 (34–73)
	30 km^2^	169 (146–204)	124 (102–151)	109 (89–138)	103 (75–133)	101 (71–125)
	50 km^2^	244 (210–273)	160 (133–206)	132 (100–158)	124 (98–149)	120 (92–143)
	70 km^2^	302 (261–349)	177 (145–202)	144 (109–169)	138 (110–178)	130 (103–160)
	91 km^2^	346 (313–377)	188 (159–222)	153 (129–182)	145 (123–163)	137 (111–154)
	111 km^2^	386 (353–417)	193 (149–222)	157 (122–179)	150 (128–175)	142 (117–169)
	131 km^2^	415 (383–443)	196 (159–224)	160 (140–191)	154 (130–168)	145 (122–175)
	151 km^2^	442 (403–475)	196 (170–233)	164 (135–187)	157 (142–171)	147 (126–168)
	171 km^2^	465 (433–496)	195 (173–229)	163 (136–185)	160 (142–180)	154 (131–172)
	191 km^2^	483 (442–512)	197 (166–231)	164 (140–183)	163 (140–188)	156 (136–173)
	201 km^2^	489 (459–512)	198 (166–229)	165 (147–189)	164 (147–182)	156 (137–173)

### 2.6 Objective 2: Spatial sampling effort for accurate population estimates

**FIGURE A2:**
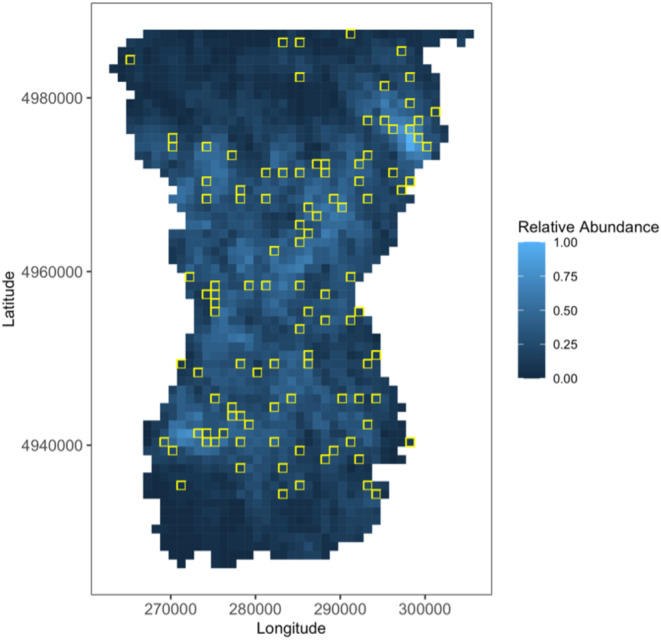
An example of the simulated spatial sampling used in this study. Each cell represents a 1 km^**2**^ area, with color values indicating the local relative abundance of moose (*Alces americanus)*. Cells were randomly selected (enclosed in yellow boxes), stratified by relative abundance values.

We quantified the spatial extent required to reach accurate population estimates using pedigree reconstruction (hereafter spatially based sampling), as a more easily quantified metric of effort compared to population‐based sampling. We “surveyed” a varying number of 1 km^2^ grid cells within a sampling grid overlaid across the study area and collected samples from individuals that used those grid cells (Figure A2). We explored a range of realistic sampling efforts, where new cells were sampled each year. We randomly sampled 10–200 grid cells annually, representing 0.06%–12.2% of the study area, with 100 sampling iterations for each level of spatially based sampling in each year. The resulting 10,000 sampling iterations represented an annual survey effort of 10–200 km^2^ (Table 2). We randomly selected grid cells for sampling, weighted by the number of moose using each grid cell to mimic prior knowledge available to wildlife practitioners (i.e., habitat suitability or occurrence data). To calculate these weights, we generated a 100% minimum convex polygon for each individual using the adehabitatHR package (Calenge, 2006), and used the over() function from the sp package (Bivand et al., 2013; Pebesma & Bivand, 2005) to calculate the number of individuals overlapping each grid cell (Figure A2). These abundances were scaled from 0 to 1 based on the grid cell with the highest abundance value.

When a grid cell was selected for sampling, we calculated the utilization probability for each moose using that pixel as a proxy for encountering scat, using their winter locations and the kde2d() function from the MASS package (Venables & Ripley, 2002). We scaled these utilization probabilities between 0 and 1 for each animal based on their highest utilization probability estimated across all grid cells and used these scaled probabilities as the probability of sampling each animal that used a sampled grid cell. In doing so, we assumed that if an individual was using a grid cell, detection was a process based on utilization, rather than the ability of a survey team to find a scat. Due to imperfect detection and stratified cell selection, total sample sizes were lower in the spatial sampling approach (Objective 2) compared to the population‐based sampling (Objective 1). As with Objective 1, each year of sampling both broadened and lengthened the estimated pedigree. We calculated precision (CV), bias (SME), accuracy (SRMSE), growth rate, and confidence intervals to evaluate the performance of the pedigree reconstruction estimator using spatial sampling.

### 2.7 Objective 3: Performance of estimator across varying local animal densities

We delineated nine, equally sized zones (298 km^2^) that spanned the area of the simulated population to test how local densities influence what sampling intensity is required. We used location data from the final year of the 25‐year population simulation to calculate the density within each zone. We then sampled 1%–20% of 1 km^2^ grid cells delineated for Objective 2 in each zone (3–60 km^2^) with the same stratified, random sampling design. We then used pedigree reconstruction to estimate population density (with 95% confidence intervals) for each zone across sampling efforts. We examined the role of population density in the required sampling effort for pedigree reconstruction to produce accurate abundance estimates and to detect changes in population size.

## 3 RESULTS

**FIGURE 4:**
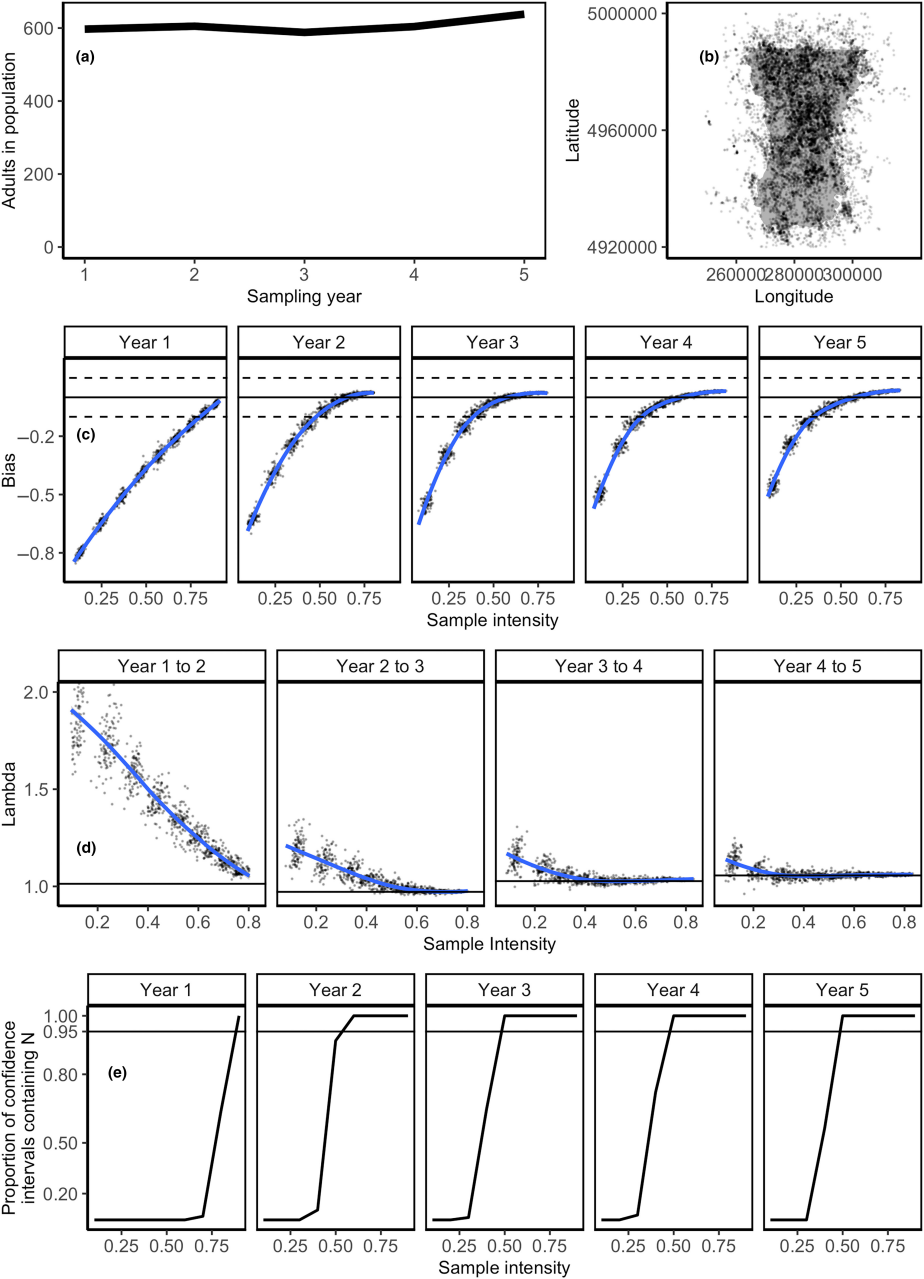
Performance of pedigree reconstruction to estimate the adult moose (*Alces americanus)* population size and trend across a range of population‐based sampling intensities. Sampling intensity refers to the proportion of unsampled individuals sampled each year. (a) The number of adults in the study area across the 5 years of sampling, after a 20‐year burn‐in period. (b) A subset of animal locations illustrating the distribution of animals across the study area. (c) The bias in pedigree reconstruction estimates (points) and the average bias across these simulations (blue lines). Solid black lines indicate no bias in a population estimate, and black dashed lines indicate 10% bias. (d) Point and average estimates of population growth rate (lambda; points and blue lines, respectively), compared to the population's growth rate (black solid line). (e) Proportion of population sampling iterations for which the 95% confidence intervals overlapped the true adult population size. Our uncertainty measure is correct when these proportions meet or exceed their expected frequency.

Our simulated moose population exhibited a variable trajectory, with our initial population of 659 moose growing very slowly to 872 moose by the end of the 25‐year simulation (*λ* = 1.011). Of these individuals in the final year of the simulation, 828 moose used some portion of the study area (Table 2). The adult segment of the population (≥2 years old) followed a similar trajectory, with an initial 517 adult moose increasing to 675 adult moose, 638 of which overlapped with the study area (Table 2; Figure 4a,b).

### 3.1 Objective 1: Accuracy of updated pedigree reconstruction

**FIGURE 5:**
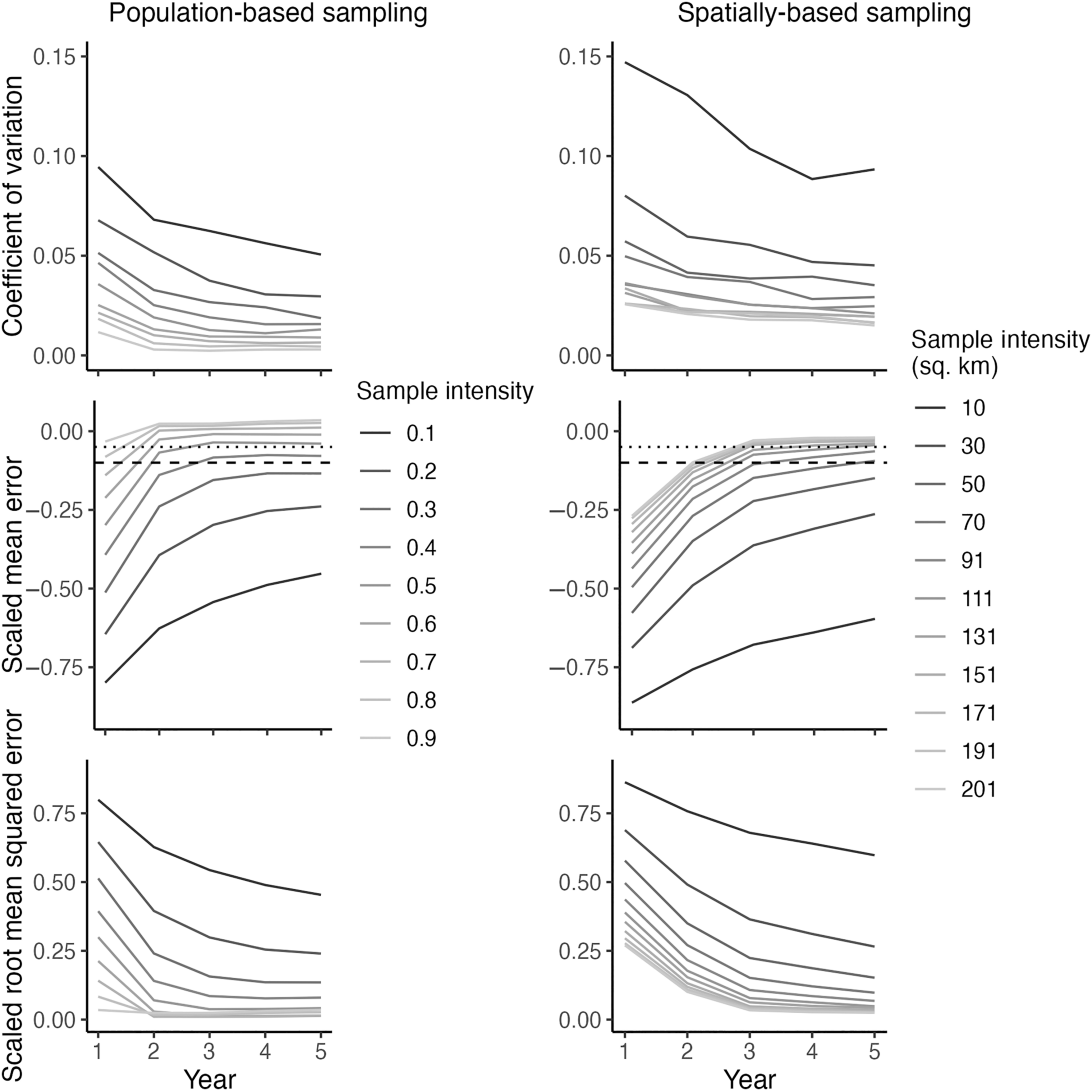
A comparison of precision, bias, and accuracy for pedigree reconstruction abundance estimates between various sampling intensities of the population (population‐based sampling) or of the study area (spatially based sampling). We quantified precision as the coefficient of variation, bias as the scaled mean error, and accuracy as the scaled root mean squared error. For reference, scaled mean error of −5% and −10% are indicated with a dotted and dashed line for each sampling scheme, respectively.

By sampling new individuals over multiple years, pedigree reconstruction provided accurate estimates of the adult moose population and its rate of change. With adequate population‐based sampling, pedigree reconstruction estimates were within 10% of the true number of adults in the population (Figures 4c and 5). As sampling effort increased within each year, estimates were asymptotic, indicating a threshold at which further sampling effort yielded little benefit (Figure 4c). Precision was high for all sampling intensities, as measured by CV, and increased for all sampling intensities as surveys continued through time, reaching under 5% for all sampling intensities (Figure 5). Sampling effort necessary for minimizing bias (SME) and maximizing accuracy in population estimates decreased as the survey period progressed (Figure 5). In the first year of sampling, estimates with low bias (> −5% scaled mean error; SME) and high accuracy (SRMSE) were only possible by sampling the majority of unsampled individuals (90%; Figure 5). This low bias threshold was met with 60% sampling by Year 2, and 50% sampling by Year 3 (Figure 5); Bias with sampling ≤40% of unsampled individuals stabilized below the −5% SME threshold, indicating that sampling at these lower levels would not reduce bias with additional sampling (Figure 5), even with a changing population size. Alternatively, with intensive population‐based sampling over the 5‐year survey, estimates were slightly biased (SME > 0) to overestimate abundance by an average of 3.5%, due to the inclusion of previously sampled adults that exited the study area through emigration or death (Figure 5).

Population growth rates derived from pedigree reconstruction (*N*_*t*+1_/*N*_*t*_) were greatly overestimated in the first 2 years of sampling, particularly when sampling effort was low (Figure 4d). As sampling progressed throughout the simulated study, broadening and lengthening the estimated pedigree, estimated growth rates became asymptotic around the true population growth rate for each interval, and the sampling effort necessary to accurately estimate population growth rate decreased. Growth rate estimates could distinguish between slight population growth (Year 3–4 and Year 4–5) and decline (higher sampling efforts, Year 2–3, Figure 4d), even in this mostly stable population.

Our proposed method to estimate uncertainty around population estimates contained the true population size with enough sampling intensity over multiple surveys. When 50% of unsampled individuals were sampled in three or more years, 95% confidence intervals overlapped the true populations size as predicted (Figure 4e). Confidence intervals for lower sampling intensities did not include the known population size as predicted (Figure 4e).

### 3.2 Objective 2: Spatial sampling effort for accurate population estimates

**FIGURE 6:**
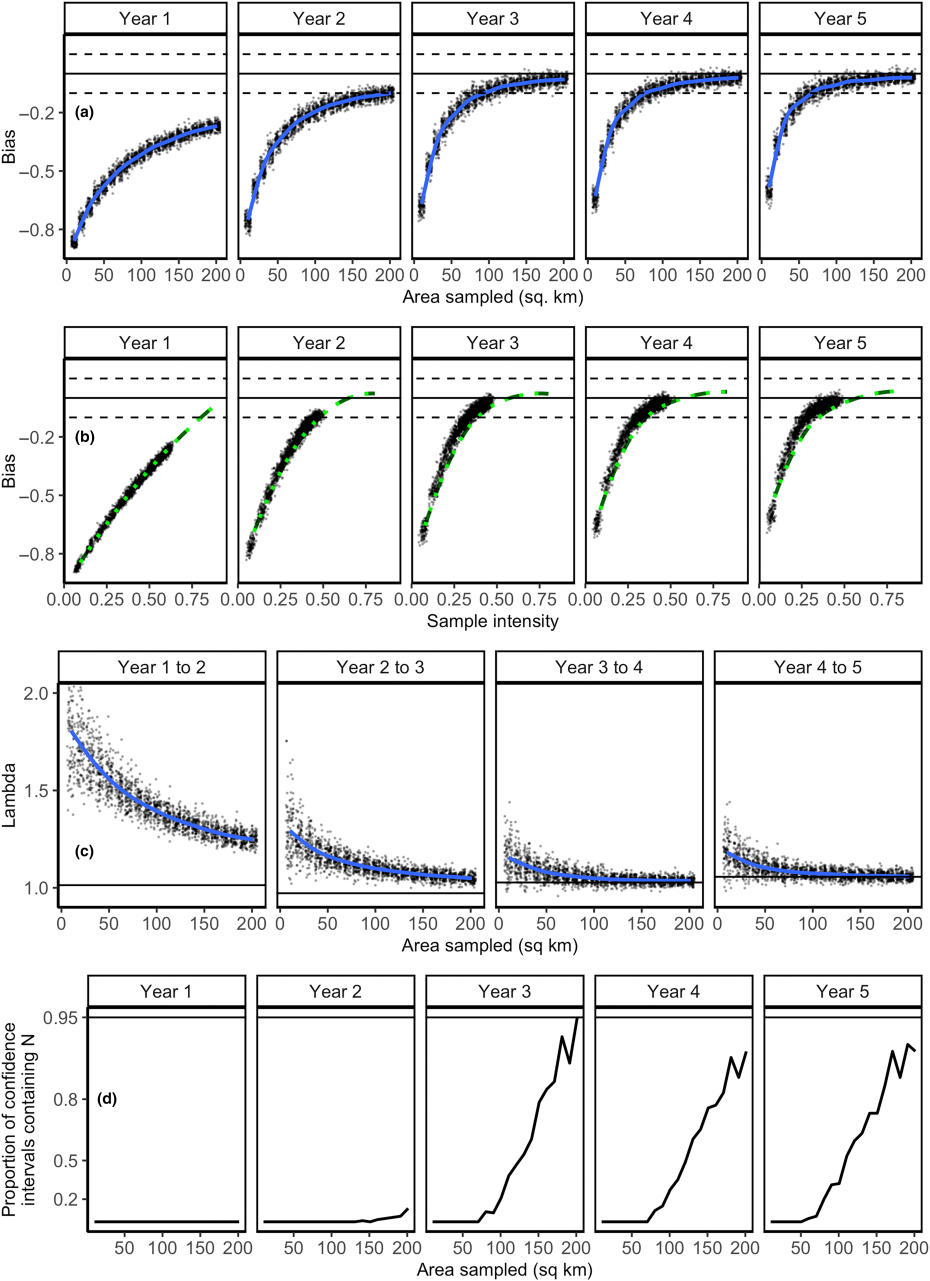
Performance of pedigree reconstruction over a range of spatial sampling intensities. Area sampled refers to the area surveyed in the study area considered. (a) The bias in pedigree reconstruction estimates using spatially based sampling (points) and the average bias across these simulations (blue lines). Solid black lines indicate no bias in a population estimate, and black dashed lines indicate 10% bias. (b) The equivalent population sampling intensities for spatial sampling efforts simulated, with comparison with mean bias from Objective 1 (green dashed line). (c) Estimates of population growth rate (lambda) across varying spatial sampling efforts. (d) Proportion of spatial sampling iterations that successfully captured the true population size within 95% confidence intervals.

Our simulated sample collection across our study area yielded patterns consistent with our population sampling scheme reported in Objective 1 while providing a more intuitive metric of effort (Figure 6a). However, precision, bias, and accuracy were lower relative to those attained by population‐based sampling (Objective 1; Figure 5). While we stratified sampling based on the numbers of moose using each grid cell, even extensive sampling efforts did not capture more than 63% of unsampled individuals (Figure 6b, Year 1), in part because the probability of sample detection was not 1 in this simulation. Therefore, our inference that spatial sampling is consistent with population‐based sampling is limited to the proportion of previously unsampled individuals sampled realized by our spatial sampling (Figure 6b). Given the assumptions and conditions of our simulated moose population and survey methods, collecting samples from moose over 100 km^2^ for 5 years would produce minimally biased estimates (SME ≥ −5%) of the total adult population (Figures 6a and 5). As with population‐based sampling, both precision and accuracy increased for all sampling intensities as surveys continued through time. Population growth rate estimates can be unbiased over time, with accurate estimates of slight population increases from substantial sampling effort (150 km^2^; Figure 6c). Our measures of uncertainty did not perform well in our spatial sampling simulation, with 95% confidence intervals not containing the true population size for 95% of simulations (Figure 6d). This underperformance is most likely the result of the limited proportion of the population sampled achieved with spatially based sampling (Figure 6b).

### 3.3 Objective 3: Performance of estimator across varying local animal densities

**FIGURE 7:**
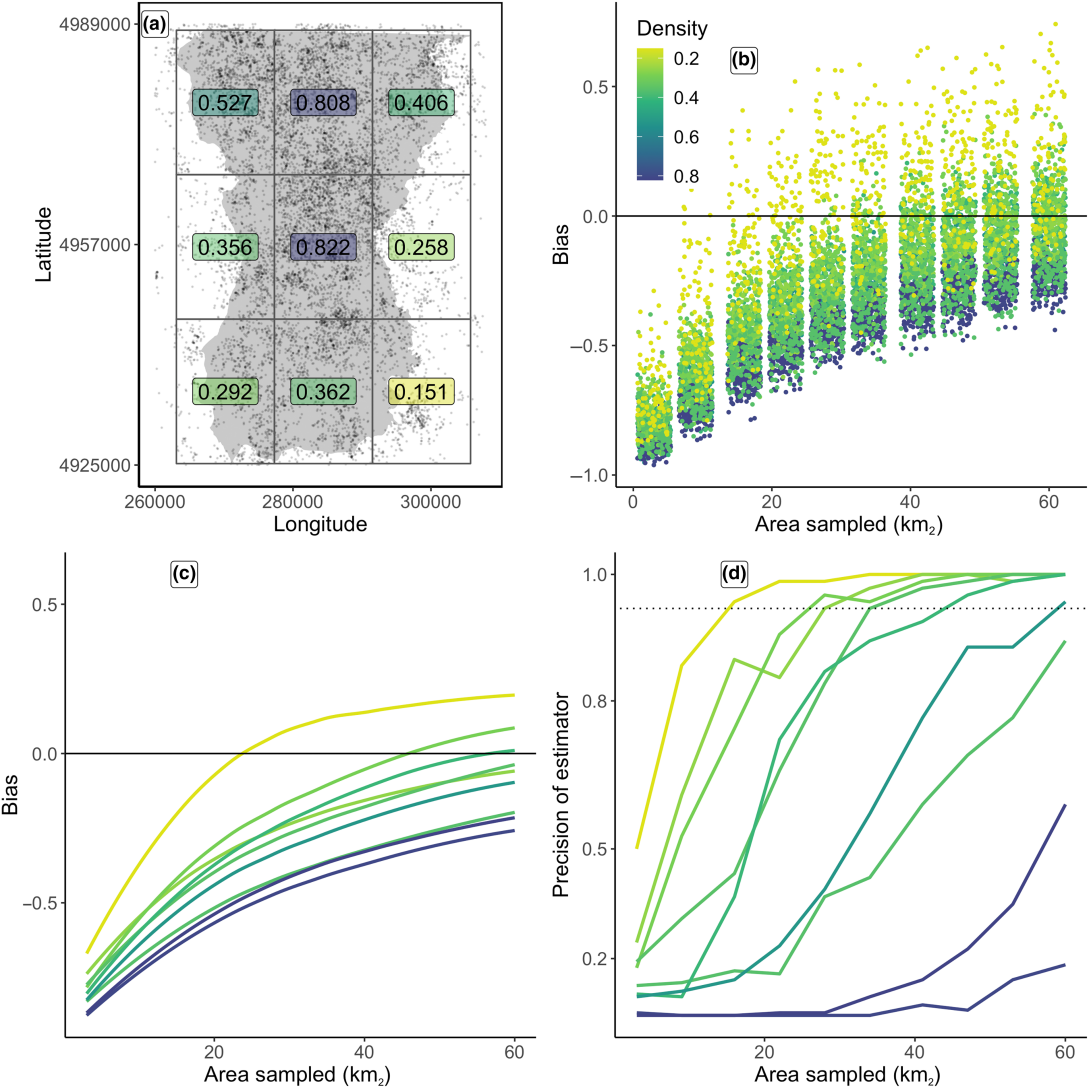
Performance of pedigree reconstruction across zones with varying local densities of a simulated moose (*Alces americanus)* population. (a) Sample collection was simulated across 3–60 km^2^ of nine 298 km^2^ localities, allowing for pedigree reconstruction and abundance estimation in each locality. (b) Bias of simulated point estimates across spatial sampling intensity and across local density. (c) Average bias of simulated point estimates. (d) Proportion of spatial sampling iterations for each locality that overlapped the true population size within 95% confidence intervals.

In the final winter of the 25‐year simulation, moose densities varied from 0.151 to 0.822 moose per km^2^ across the nine 298 km^2^ zones delineated for this objective (Figure 7a). For perspective, the simulated density for the entire study area was 0.528 moose per km^2^. There was a clear, positive relationship between density and the level of sampling effort required for unbiased estimates of population size as estimated by 1‐year pedigree reconstruction as density increases (Figure 7b,c). Importantly, the total area to be sampled with low bias depended on the zone's density: oversampling low‐density zones produced positively biased population estimates, while undersampling high‐density zones produced negatively biased population estimates. However, the bootstrapped confidence intervals were sufficiently large enough to capture the true population estimate such that as sampling effort increased, 100% of the bootstrapped intervals contained the true population size (Figure 7d). The spatial sampling required for 95% confidence intervals to overlap true abundance with ≥95% frequency increased with local density, yet 95% confidence intervals for the highest local densities did not overlap true local abundance with ≥95% frequency (Figure 7d).

## 4 DISCUSSION

We improved pedigree reconstruction as an abundance estimator by integrating a probabilistic approach that produces accurate estimates of abundance and annual growth and provides metrics of uncertainty around these estimates. We demonstrated the use of stratified, random sampling across a discrete area as an approach without requiring some prior knowledge of the population's size (Larroque & Balkenhol, 2023). Accurate estimates of population size and annual growth were attainable for an entire population only with sufficient sampling over multiple years, yet this required effort decreased when considering smaller localities and low population densities. This study provides a key step in further developing pedigree reconstruction as a reliable population estimator to be added to existing approaches for estimating abundance (Larroque & Balkenhol, 2023).

Our results indicate that pedigree reconstruction may be an appropriate estimation tool for low‐density, small populations, particularly for rare and endangered species. These conditions may prove difficult for other genetic abundance measures such as genetic mark–recapture methods, which rely on multiple samples from each individual (Puechmaille & Petit, 2007), and close‐kin mark–recapture methods, which rely on sampling harvested individuals (Bravington et al., 2016; Marcy‐Quay et al., 2020). Our selection of moose as a test species, along with testing the role of local density on accuracy, demonstrates this. When we considered the population (*n* = 828 moose, 638 adults in the final year of the simulation), pedigree reconstruction produced accurate estimates after multiple years of intensive sampling. However, when the population was broken into nine localities varying in moose density, pedigree reconstruction could estimate population size within the lower density segments with much less effort (though with a cost in precision) in only one survey. This is likely due to a smaller pedigree to reconstruct, requiring fewer individuals be sampled than in a larger population of similar density. We present our findings with caveats that our inferences are based on this simulated moose population, and that inferences may differ for other species with other life history characteristics and reproductive strategies.

Pedigree reconstruction estimates abundance beyond the discrete area, jurisdiction, or protected area where sampling occurs, leading to positive bias in abundance estimates for discrete areas of interest. This bias is likely the result of inferred and linked individuals that either leave the population through emigration or mortality, especially if the pedigree is reconstructed with samples that are combined over years. However, this positive bias appears to be small, predictable, and easily corrected for. Species life history, dispersal, and reproductive behavior may be contributing factors in the bias of pedigree reconstruction estimates.

A key assumption behind pedigree reconstruction is that the probability of being sampled and the probability being matched to an offspring are independent of each other. This method relies on observed conditional probabilities *p*(sampled|matched) and *p*(matched|sampled) to estimate the marginal probabilities *p*_sampled_ and *p*_matched_, respectively (Appendix 1: A2). Matched and unmatched individuals may have different probabilities of being sampled if a species' life history leads to matched adults being easier to sample than unmatched individuals (or vice versa). In our simulated study, if an adult female or a calf were included in the sample, both individuals were sampled. However, the simulated means of sample collection, where a surveyor intersects tracks and samples from an individual in snow, likely reduces any increased probability of sampling matched adults over sampling unmatched adults. Study designs must consider seasonal or life stage behaviors that could make sampling unmatched individuals more difficult than sampling matched individuals. Sampled and unsampled individuals may have different *p*_matched_ if there is any bias in sampling their offspring. Study designs must ensure that sampling is distributed to ensure the sampling of offspring that can be matched to both sampled adults (linked) and unsampled adults (inferred; Appendix 1: A1). Knowledge of a species' dispersal behavior could improve study design by minimizing the risk of sampling offspring only from sampled adults.

Our simulated, spatially explicit moose population allowed us to develop an alternate metric of sampling effort, one based on surveyed area rather than sampled individuals. This easily quantifiable approach maximizes the number of new individuals sampled in each survey while minimizing the costs of unknowingly resampling animals. Practitioners often seek a balance between maximizing the number of animals sampled and the spatial extent of sampling for an array of survey methods (Boulanger et al., 2004; Sun et al., 2014). This trade‐off is also important for pedigree reconstruction, and while local densities are usually not known, proxies based on expert and local knowledge or general understanding of habitat suitability could inform this survey approach (Kuhnert et al., 2010; Pearman‐Gillman et al., 2020).

Pedigree reconstruction can be a cost‐effective estimator if the probability of detecting an individual is maximized. In this context, the detection process refers to encountering genetic material on the landscape. In this simulated study, high levels of spatially based sampling (200 km^2^) after 3 years yielded an equivalent population‐based sampling rate of under 50% sampling intensity. While this sampling rate produced accurate estimates of population size, this effort is likely greater than what practitioners can adopt. This shortcoming may have resulted from our use of utilization distribution values as a proxy for the probability of collected samples in a surveyed grid cell. Our coarse survey unit (1 km^2^) did not capture variation in finer scale utilization (Boyce, 2006), and we likely underestimated the probability of an animal using this spatial unit. We also did not consider aggregations of moose, occurring in areas with favorable microclimate conditions, forage availability, and vegetative cover (Peek et al., 1974). By increasing detection of individuals by designing finer scale surveys and benefiting from social groups or clumped resource availability, pedigree reconstruction may produce accurate estimates at lower survey efforts.

A key consideration for the use of pedigree reconstruction is the cost of collecting and genotyping genetic samples. Pedigree reconstruction does not rely on multiple samples from individuals, unlike the sampling intensity required for genetic mark–recapture (Puechmaille & Petit, 2007). We found that sampling 60%, 50%, and 40% of previously unsampled individuals over 2, 3, and 4 years, respectively, yielded accurate estimates. On average, these efforts sampled 769, 772, and 793 animals, respectively. Using the genotyping costs of 22 USD per sample for concurrent moose genetic studies (Rosenblatt, Pers. Comms), this sampling effort cost between 16,918 and 17,446 USD. Regardless of how sampling efforts are spread across years, inadequate sampling effort may yield biased and imprecise estimates. Practitioners may save funds by investing in shorter survey periods to minimize personnel and logistical costs, as longer surveys may require greater investment through time. Further savings could be made if sampling focused on smaller areas, where local densities are of concern. Importantly, applications could minimize the risk of resampling of previously sampled individuals to minimize overall costs. Resampling could be reduced if spatially based sampling was not only stratified by relative abundance or utilization, but also spaced to ensure sampling was not clustered within the home ranges of a limited subset of the population. If resampling remains a challenge and there are suitable recaptures of individuals to also estimate abundance with genetic CMR approaches, multiple estimates from both pedigree reconstruction and genetic CMR methods would prove useful.

Two issues remain with the application of pedigree reconstruction beyond a simulated application provided here, including genotyping error and detection of related individuals beyond parent–offspring relationships over multiple surveys. We assumed clear, parent–offspring linkages between related individuals in this simulation study but acknowledge that both issues can add uncertainty into abundance estimates. Recent works that utilize SNP arrays for wildlife species of concern have demonstrated approaches that reduce genotyping errors and reduce false detections of parent–offspring relationships (Ekblom et al., 2021). Future applications of pedigree reconstruction to estimate abundance could benefit from rapidly advancing genotyping and documenting efforts to reduce genotyping and relationship assignment errors.

### 4.1 Future applications

Designing a sampling strategy for pedigree reconstruction depends on the life history and behavior of a target species. Practitioners may benefit from documenting demographic and spatial information, along with individual identity prior to sampling to establish relationships and avoid future resampling (Creel & Rosenblatt, 2013; Spitzer et al., 2016). Practitioners might maximize the number of animals sampled when designing sample collection, focusing on sampling when a species aggregates, across a broad spatial extent. spatially based sampling approaches could help meet this challenge, and reduce costs from unnecessary resampling of individuals.

Several examples of potential applications of pedigree estimation illustrate its future use. The African lion is individually identifiable (Pennycuick & Rudnai, 1970), highly social, and concentrate around the areas of high prey density in groups (Schaller, 1972). Lion prides and coalitions remain in a single place for hours or days after a successful hunt, allowing for fecal sample collection of multiple individuals around the kill site (Tambling et al., 2012; Tambling & Belton, 2009). Additionally, sampling dispersing individuals aids in the inference of parents in populations inhabiting inaccessible areas, as noted by Shimozuru et al.'s (2022) study of brown bears. Alternatively, low‐density populations with restricted ranges would be suitable for pedigree reconstruction. Low‐density moose populations exist along the southern edge of the species distribution in eastern North America. Suitable habitat for these moose population are often known (e.g., Blouin et al., 2021a, 2021b) and could inform spatially based sampling for pedigree reconstruction and aid in the monitoring of low‐density populations.

More applications of pedigree reconstruction with wild populations would further advance this novel approach. With the developments of the pedigree reconstruction approach in this study, studies may be able to report a measure of precision for their population estimates and use successive sampling across time to accurately estimate the conditional probabilities core to pedigree reconstruction. Additionally, the practical reconstruction of a pedigree comes with some uncertainty around the most probable pedigree, which could be integrated into the uncertainty around the probabilities underpinning pedigree reconstruction and resulting estimates. Well‐studied populations of easily sampled, social species could easily demonstrate and advance the application of this method to be used with poorly studied populations of conservation concern.

## AUTHOR CONTRIBUTIONS

**Elias Rosenblatt:** Conceptualization (lead); formal analysis (lead); investigation (lead); methodology (equal); software (lead); visualization (lead); writing – original draft (equal). **Scott Creel:** Conceptualization (supporting); writing – review and editing (equal). **Katherina Gieder:** Supervision (supporting); writing – review and editing (equal). **James Murdoch:** Supervision (supporting); writing – review and editing (equal). **Therese Donovan:** Conceptualization (equal); formal analysis (equal); funding acquisition (equal); methodology (equal); project administration (equal); supervision (equal); writing – review and editing (equal).

## CONFLICT OF INTEREST STATEMENT

None stated.

## Data Availability

Simulation R scripts and datasets written by E.R. are available in a github repository (https://github.com/erosenbl/pedigreeReconstruction.git).

## APPENDIX 1

### 1.1

*A1*: The premise behind pedigree reconstruction as an estimator of population size. Individuals in a population belong to one of four mutually exclusive and exhaustive states, which sum to the total adult population size. Two characteristics dictate which of these population segments an individual belongs to, including whether the individual is genetically sampled during a survey (with two outcomes: sampled or unsampled), and whether that individual is matched to an offspring within the pedigree (with two outcomes: matched or unmatched). Four combinations of these two characteristics result in the following matrix:

**Table:**

	Sampled (S)	Not sampled (~S)	Marginals
Matched (M)	S,M = matched to offspring and sampled = *N*_linked_	~S,M = matched to offspring but not sampled = *N*_inferred_	*N*_matched_
Not matched (~M)	~S,M = sampled but not matched to offspring = *N*_unlinked_	~S,~M = not sampled and not matched to offspring = *N*_invisible_	*N*_unmatched_
Marginals	*N*_sampled_	*N*_unsampled_	*N*_total_

where: *N*_linked_ = sampled individuals identified as a parent to another sampled individual(s). *N*_unlinked_ = sampled individuals unrelated to other sampled individuals. *N*_inferred_ = unsampled individuals inferred to exist with sampling of offspring and mate. *N*_invisible_ = unsampled individuals invisible to pedigree reconstruction.

An estimate of the total population is the sum of the four conditions:

(1)
$$N_{\text{total}}=N_{\text{linked}}+N_{\text{unlinked}}+N_{\text{inferred}}+N_{\text{invisible}}.$$

The fourth term is unobserved. However, the total number of sampled individuals is *N*_linked_ + *N*_unlinked_, and the total number of confirmed individual parents is *N*_linked_ + *N*_inferred_. The number of adults in any of the four states in the above table can be corrected by the joint probability of being in a given state, calculated as the product of two conditional probabilities from the pedigree data. For example, the two conditional probabilities of being a linked adult are as follows:

(2)
$$p\left(S|M\right)=\frac{N_{\text{linked}}}{N_{\text{linked}}+N_{\text{inferred}}}$$

(3)
$$p\left(M|S\right)=\frac{N_{\text{linked}}}{N_{\text{linked}}+N_{\text{unlinked}}}.$$

Under the key assumption that the probability of being sampled is independent from the probability of being matched, an estimate of the total population *N*_total_ is

(4)
$$P_{\text{linked}}=p\left(S|M\right)*p\left(M|S\right)$$

(5)
$$N_{\text{total}}=\frac{N_{\text{linked}}}{P_{\text{linked}}}.$$

*A2*: An example of deriving the conditional, marginal, and joint probabilities at the core of pedigree reconstruction. Recalling from Appendix 1: A1, individuals exist in one of four segments (in bold):

**Table:**

Condition	Sampled	Unsampled	Marginals
Matched	***N***_**linked**_	***N***_**inferred**_	*N*_matched_
Unmatched	***N***_**unlinked**_	***N***_**invisible**_	*N*_unmatched_
Marginals	*N*_sampled_	*N*_unsampled_	*N*_total_

Marginal and joint probabilities can be estimated from these frequencies, which allow adult population estimates:

**Table:**

Condition	Sampled	Unsampled	Marginals
Matched	***p***_**linked**_	***p***_**inferred**_	*p*_matched_
Unmatched	***p***_**unlinked**_	***p***_**invisible**_	*p*_unmatched_
Marginals	*p*_sampled_	*p*_unsampled_	1.00

Under the assumption that the sampling and matched are independent characteristics, *p*(sampled|matched) = *p*(sampled), and *p*(matched|sampled) = *p*(matched). Assuming a beta prior distribution with hyperparameters *α* and *β* can be established for each *p*_sampled_ and *p*_matched_, the binomial successes and failures from the observed pedigree data can be used to update these hyperparameters using the Bayesian conjugate solutions, providing a posterior beta distribution that reflects the current knowledge of *p*(sampled) and *p*(matched):

**Table:**

$\alpha_{\text{sampled}}^{\prime }=\alpha +N_{\text{linked}}$	Equation 5	$\alpha_{\text{matched}}^{\prime }=\alpha +N_{\text{linked}}$	Equation 6
$\beta_{\text{sampled}}^{\prime }=\beta +N_{\text{inferred}}$	Equation 7	$\beta_{\text{matched}}^{\prime }=\beta +N_{\text{unlinked}}$	Equation 8

In this application, prior distributions are vague, with no prior knowledge influencing these error distributions. The posterior distributions (beta distributions) provide a means of incorporating uncertainty into the pedigree reconstruction (Fink, 1997), as random values of *p*(sampled) and *p*(matched) can be drawn from each distribution in a bootstrap simulation, and *N‐hat* is calculated in Appendix 1: A1. Confidence intervals on *N* can be obtained by summarizing the bootstrap results.
